# Oral Glutamine Supplementation Reduces Obesity, Pro-Inflammatory Markers, and Improves Insulin Sensitivity in DIO Wistar Rats and Reduces Waist Circumference in Overweight and Obese Humans

**DOI:** 10.3390/nu11030536

**Published:** 2019-03-01

**Authors:** Kahlile Youssef Abboud, Sabrina Karen Reis, Maria Eduarda Martelli, Olivia Pizetta Zordão, Fabiana Tannihão, Alessandra Zanin Zambom de Souza, Heloisa Balan Assalin, Dioze Guadagnini, Guilherme Zweig Rocha, Mario Jose Abdalla Saad, Patricia Oliveira Prada

**Affiliations:** 1 School of Applied Sciences, State University of Campinas (UNICAMP), Limeira 13484-350 SP, Brazil; kahlilebbd@gmail.com (K.Y.A.); reis.sabrinakaren@gmail.com (S.K.R.); mariaeduardamartelli@hotmail.com (M.E.M.); ftannihao@yahoo.com.br (F.T.); alessandrazz@hotmail.com (A.Z.Z.d.S.); 2 Department of Internal Medicine, State University of Campinas (UNICAMP), Campinas 13083-887 SP, Brazil; oliviapizetta@hotmail.com (O.P.Z.); helo_assalin@yahoo.com.br (H.B.A.); diozeg@gmail.com (D.G.); gzrocha@gmail.com (G.Z.R.); msaad@fcm.unicamp.br (M.J.A.S.)

**Keywords:** obesity, glutamine supplementation, inflammation, insulin sensitivity, cytokines, clamp, LPS, hexosamine

## Abstract

In the present study, we aimed to investigate whether chronic oral glutamine (Gln) supplementation may alter metabolic parameters and the inflammatory profile in overweight and obese humans as well as whether Gln may modulate molecular pathways in key tissues linked to the insulin action in rats. Thirty-nine overweight/obese volunteers received 30 g of Gln or alanine (Ala-control) for 14 days. Body weight (BW), waist circumference (WC), hormones, and pro-inflammatory markers were evaluated. To investigate molecular mechanisms, Gln or Ala was given to Wistar rats on a high-fat diet (HFD), and metabolic parameters, euglycemic hyperinsulinemic clamp with tracers, and Western blot were done. Gln reduced WC and serum lipopolysaccharide (LPS) in overweight volunteers. In the obese group, Gln diminished WC and serum insulin. There was a positive correlation between the reduction on WC and LPS. In rats on HFD, Gln reduced adiposity, improved insulin action and signaling, and reversed both defects in glucose metabolism in the liver and muscle. Gln supplementation increased muscle glucose uptake and reversed the increased hepatic glucose production, in parallel with a reduced glucose uptake in adipose tissue. This insulin resistance in AT was accompanied by enhanced IRS1 O-linked-glycosamine association in this tissue, but not in the liver and muscle. These data suggest that Gln supplementation leads to insulin resistance specifically in adipose tissue via the hexosamine pathway and reduces adipose mass, which is associated with improvement in the systemic insulin action. Thus, further investigation with Gln supplementation should be performed for longer periods in humans before prescribing as a beneficial therapeutic approach for individuals who are overweight and obese.

## 1. Introduction

Obesity has become a significant public health problem worldwide, and it is associated with various comorbidities [1,2,3,4,5]. Obesity is considered a low-grade inflammatory disease and the degree of inflammatory status correlates positively with the development of insulin resistance and type 2 diabetes mellitus [6,7,8]. The white adipose tissue has a primary role sensing and managing energy homeostasis [7]. Consistently, human and rodents studies demonstrated that, under a positive energy balance, the white adipose tissue triggers an immune response, which develops low-grade inflammation milieu, associated with infiltration of immune cells [7,8,9]. Additionally, products from intestinal microbiota as lipopolysaccharides (LPS) can also contribute to this inflammatory state in obesity [10,11,12].

Many efforts have been made to prevent and treat obesity and to reduce the low-grade inflammatory status, including hypocaloric diets combined or not combined with physical activity and drugs in humans [13]. However, the complexity and time spent in this treatment often lead to weight recovery [14]. Thus, it will be useful if an obesity treatment strategy takes into account single nutrient supplementation. Diets with glutamine (Gln) supplementation have aroused interest since they can mitigate the release of cytokines, reduce organ damage, and improve survival of mice and humans with endotoxemia [15,16,17,18,19,20]. However, studies that evaluated the potential of oral glutamine supplementation decreasing body weight and fat mass in an obese human being are scarce [21,22,23].

Chronic oral glutamine supplementation was shown to improve fasting blood glucose and A1c as well as reduced body fat and waist circumference (WC) in type 2 diabetic individuals [23]. In another study, oral glutamine supplementation for four weeks was able to reduce body weight (BW) and WC, but not insulinemia and HOMA-IR in overweight and obese female patients [22]. However, none of these studies investigated possible pathophysiological mechanisms of how glutamine supplementation could contribute to weight and adipose mass reduction and improve metabolic parameters.

Insulin has a potent lipogenic effect on adipose tissue [7]. This effect is related to the tyrosine phosphorylation of the insulin receptor by insulin, which induces PI3K/Akt pathway activation that leads to glucose transport, and lipogenesis afterwards [7,8]. Mice with specific insulin receptor disruption on fat (FIRKO mice) are protected from obesity induced by a high fat diet [24]. On the other hand, animals on the high fat diet usually displayed insulin resistance in the liver and muscle but not in the adipose tissue [25,26]. These data argue that, in some specific conditions, the lack of insulin effects in adipose tissue may protect from obesity and some of its comorbidities. Previous cell culture studies suggested that glutamine was able to reduce insulin action in adipocytes, but not on L6 muscle cells [27,28,29]. Accordingly, the lack of effect of insulin on the adipose tissue through glutamine supplementation might be beneficial in reducing lipogenesis and, hence, fat accumulation in vivo.

Thus, we combined human and animal models to deeper understand the mechanisms by which glutamine may reduce obesity and its comorbidities. Herein, we aimed to investigate (1) whether chronic oral Gln supplementation may alter anthropometric, metabolic parameters and also the inflammatory profile in overweight and obese humans in a proof of concept study. We also attempted to investigate (2) whether chronic Gln supplementation via gavage may alter these same parameters in high-fat diet (HFD) rats, which are integrated with an investigation of insulin action and signaling in specific tissues as liver, muscle, and adipose tissue. We attempted to understand, at a molecular level, this beneficial effect of glutamine.

## 2. Materials and Methods

### 2.1. Human Study

This study was conducted by the Declaration of Helsinki (1964) and was approved by the Ethics Committee of the Department of Internal Medicine at State University of Campinas (UNICAMP), Campinas, SP, Brazil. All the subjects gave written informed consent.

All volunteers were employees with a majority of nurses at the Sumare State Hospital in Sumare city, São Paulo state, Brazil. A total of 150 volunteers were randomly recruited via advertisements placed around the hospital. In this double-blind study, 39 volunteers completed the intervention period. The inclusion criteria to participate were as follows: men or women adults aged between 20 and 60 years old and diagnosed as overweight or obese. Subjects who reported renal or thyroid disease, pregnancy, taking an antidepressant, and anorectic or laxative drugs were excluded from the study. Before beginning the study, body weight and height were measured using a Filizola scale with a calibrated digital scale and stadiometer, respectively (PL 200 model). The Body Mass Index (BMI) (kg/m^2^) was calculated as weight (kg) divided by height (m^2^) squared. Only overweight (BMI ≥ 25 kg/m^2^) and obese (BMI ≥ 30 kg/m^2^) volunteers were included in the study (WHO, 2000).

On the first day, volunteers were random divided into four groups according to the BMI and the supplement that they would receive: Overweight Alanine (Ala), Obese Ala, Overweight Glutamine, and Obese Gln. Afterward, blood samples were collected and waist circumference was measured. The volunteers received a kit containing small packs with 15 g of amino acid (Gln or Ala) each. They were instructed to take two packs per day, taking a total of 30 g of amino acids per day. The supplementation lasted for 14 days. The volunteers were instructed to mix the pack content in a cup of water (200 mL) before drinking and maintain the same levels of physical activity and the same diet during the 14 days of supplementation. The second measurement was done 15 days after the supplementation started. Fasting volunteers came to the hospital for the second time for BW and WC measurements and blood sample collections.

Biochemical Analysis was conducted by blood samples, which were obtained before and after the supplementation from the same volunteer. Overnight fasted volunteers had blood samples collected into tubes placed on ice. After collection, blood samples were immediately centrifuged at 1500 rpm for 15 min at 18 °C using a Centrifuge Biofuge Stratos (Hereaus, Dijkstra Vereenigde, Lelystad, Netherlands). The serum obtained was separated and transferred into 2 mL Eppendorf and stored at −80 °C until analysis. Glucose concentration was determined using the Glucose Liquiform Test (Labtest, Brazil) that applied the glucose oxidase method. All the other assays were quantified by the specific commercial enzyme-linked immunosorbent assay (ELISA). The human insulin (EZHI-14K) kit was from Millipore^®^, St. Charles, Missouri, United States. Human TNF-α (DTA00C), human IL-1β (DLB50), and human IL-6 (D6050) kits were from R&D Systems Inc., Minneapolis, MN, USA. To determine serum lipopolysaccharides (LPS) levels, the Limulus Amebocyte Assay from Cambrex (LAL kit endpoint-QCL-1000, Lonza, Walkersville, MD, United States) was used. Analyses were performed by following the specific instructions for each manufactory. HPLC system/SCL-10avp (Shimadzu Scientific Instruments, Columbia, MD, USA) and the CLASS-VP 6.12 software Class VP were used to measure serum glutamine and alanine [30] levels in order to assess the adherence to glutamine or alanine supplementation. The serum was collected before and after supplementation.

To assess the caloric intake of the individuals, we applied a 24-h food record before and after the supplementation. For analysis of the 24-h food record, we used the software Diet Pro 4.0 (Viçosa, Brazil). To assess physical activity, subjects were informed of their physical activity before and after supplementation during an interview.

### 2.2. Animal Study

Male Wistar rats received a chow diet from weaning until eleven-weeks-old. Then the rats were divided into two groups with similar body weight as follows: 1) rats that will continue receiving chow diet (chow) with 70% calories from carbohydrate, 20% from proteins, and 10% from fat, and 2) rats that start to receive a high-fat diet with 55% calories from fat, 29% from carbohydrates, and 16% from proteins. After four weeks on these diets, the rats fed with a high-fat diet were divided into two other groups. One group received glutamine supplementation (HF+Gln) and the second group received alanine supplementation (HF+Ala) for four more weeks. All diets and water were ad libitum offered. Both supplements (alanine and glutamine) were given in the drinking water (35 mL), and were prepared and replaced every day (4%). In addition, a gavage was done with alanine or glutamine (0.4 g in 1 mL) three days a week for four weeks. In accordance with Reagan-Shaw et al. [31], the ratio of conversion from human to rat studies is around six. In this regard, we used a dose of ~0.4 g/kg of glutamine in humans and ~2.4 g/kg in rats.

Body weight was followed for eight weeks. At the end of the study, the epididymal, retroperitoneal, and mesenteric fat pads were weighed.

Food was withdrawn 12 h before the blood samples collection, and the serum glucose, insulin, TNF-α, and IL-6 levels were determined by ELISA using specific kits from Linco (St Charles, MO, USA; Pierce, Rockford, IL, USA). Serum glutamine levels were determined as described before [30].

The measurement of oxygen consumption/carbon dioxide production and the respiratory exchange ratio (RER) were completed in fed rats by an indirect open circuit calorimeter (Oxymax Deluxe System, Columbus Instruments, Columbus, OH, USA), as previously described [32].

Rats were deeply anesthetized, and, as soon as the loss of pedal and corneal reflexes, the abdominal cavity was opened, and saline (200 μL) or insulin (2 μg in 200 μL) were injected in the exposed portal vein. A fragment of liver was removed after 30 s, and gastrocnemius and the epididymal fat pad were removed after 90 s. Fragments of tissues were homogenized immediately in extraction buffer, as described elsewhere [25]. The samples were then centrifuged at 15,000 rpm and 4 C for 40 min to remove insoluble material, and the supernatants were used for immunoblotting, as previously described [25,33,34] by using the following antibodies: *O*-linked N-acetylglucosamine, (MA1-072 from Affinity Biologicals, Ancaster, Ontario, Canada), Insulin Receptor Substrate 1 (IRS-1) (C-20) (sc-559), and phosphorylated protein kinase B (p-AKT-1/2/3) (Ser 473) (sc-7985-R) from Santa Cruz Biotechnologies (Dallas, TX, USA).

The hyperinsulinaemic-euglycemic clamp was performed using a previous protocol [25]. In summary, after five hours of fasting, rats were anesthetized, and two catheters were inserted including one into the left jugular vein (tracer infusion) and the other into the carotid artery (blood samples). The hyperinsulinemic-euglycemic clamp study was conducted with a continuous insulin infusion up to 120 min (rate of 3.6 mU/kg body weight per minute) to raise the concentration of insulin in plasma for approximately 800–900 pmol/L. Every 5 min, the blood samples were collected for measuring blood glucose and 10% unlabeled glucose at variable rates was infused, to maintain the glucose concentration at fasting levels. To estimate the insulin-stimulated whole-body glucose flux, a prime continuous HPLC-purified [3-^3^H] glucose (10 μCi boluses, 0.1 μCi/min) was also infused. A bolus (10 μCi) of 2-[^14^C]DG1 was infused at 90 min of the clamp to estimate skeletal muscle and visceral fat insulin-stimulated glucose-transport activity and metabolism. Blood glucose was determined at 80, 90, 100, 110, and 120 min after the clamp procedure starts for plasma [^3^H] glucose and 2-[^14^C]DG1 concentration measurements afterwards. At the end of 120 min, all rats were euthanized by anesthesia i.v. injection. The gastrocnemius skeletal muscles from hind limbs and the epididymal fat pad were taken within 2 min. All tissues were weighted, frozen with liquid nitrogen, and stored at −80 °C for future analysis.

#### Determination of the Nuclear Factor Kappa B (NF-κB) Activation

NF-κB p50 activation was determined in nuclear extracts from muscle and adipose tissue by ELISA (89858, Thermo Fisher Scientific Inc., Rockford, IL, USA), according to the recommendations of the manufacturer.

### 2.3. Statistical Analysis

The human data were collected before and after Gln and Ala supplementations and were analyzed in separated subgroups, according to BMI (overweight and obese) by using the Student’s *t* test paired two-tailed for parameters with a normal distribution or the Wilcoxon test paired two-tailed for parameters with a nonparametric distribution. A correlation between WC and LPS was made by using the Pearson test.

The animal study included three different groups, which were analyzed by One-Way ANOVA with the Bonferroni post-test. In both human and animal studies, the level of significance adopted was *p* < 0.05.

## 3. Results

### 3.1. Glutamine Supplementation in Overweight and Obese Subjects Reduce Waist Circumference and Circulating LPS

Thirty-nine adult volunteers who are overweight or obese (3 men and 36 women) were included and completed in the study protocol. They were 38.3 ± 7.2 years old. The only volunteers used in the study were the ones who maintained similar physical activity. The caloric intake of the individuals was recorded for 24 h, and only the volunteers that maintained similar caloric intake were used in the study. Volunteers did not display differences in caloric intake before and after supplementations (Overweight volunteers supplemented with Ala Before 2298 kcal/day ± 542.1 *n* = 8. After 2343 kcal/day ± 327.3 *n* = 8. Obese volunteers were supplemented with Ala before 1552 kcal/day ± 490.7 *n* = 7. After 1545 kcal/day ± 317.9 *n* = 7. Overweight volunteers were supplemented with Gln before 2069 kcal/day ± 1281 *n* = 11 and after 1923 kcal/day ± 624.7 *n* = 11. Obese volunteers were supplemented with Gln before 1816 kcal/day ± 470.6 *n* = 13 and after 2025 kcal/day ± 567.5 *n* = 13).

Data from overweight volunteers are presented in Table 1 and Table S1. Data from obese volunteers are presented in Table 2 and Table S2. No differences were observed in the body weight and BMI after supplementation with Gln or Ala in overweight volunteers (Table 1). However, the glutamine supplementation induced a significant decrease in the waist circumference of overweight subjects (Table 1). Serum glucose, insulin levels, IL-1β, IL-6, and TNF-α levels were not different after glutamine or alanine supplementation (Table 1). However, the supplementation with glutamine, but not with alanine, reduced circulating LPS levels in overweight subjects (Table 1). In addition, Ala levels were increased in Ala supplemented volunteers and Gln levels were also increased in Gln supplemented volunteers (Overweight volunteers supplemented with Ala Before 448.09 µmol/L ± 52.3 *n* = 8. After 554.46 µmol/L ± 57.64 *n* = 8. Overweight volunteers supplemented with Gln Before 419.88 µmol/L ± 35.11 *n* = 11. After 531.09 µmol/L ± 36.52 *n* = 11 and Tables S1 and S2).

**Figure 1:**
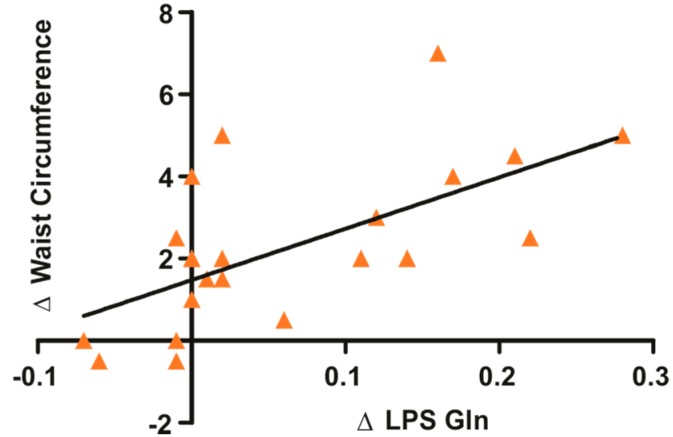
Positive correlation between the reduction of waist circumference (WC) and serum lipopolysaccharide (LPS) of obese and overweight humans. The Pearson Correlation test was used (r = 0.629).

Regarding the obese group, no differences were observed in body weight and BMI after supplementation with Gln or Ala. In a similar fashion with overweight subjects, supplementation with glutamine induced a significant reduction in waist circumference, whereas Ala supplementation did not alter this parameter (Table 2). No change in glycemia was observed in obese individuals after Gln or Ala supplementation (Table 2). However, a significant reduction in insulin levels was found in obese subjects after Gln supplementation (Table 2). No changes were observed in serum levels of TNF-α, IL-1β, and IL-6, but LPS showed a nonsignificant reduction after supplementation with Gln in obese subjects. In addition, Ala levels were increased in Ala supplemented volunteers and Gln levels were also increased in Gln supplemented volunteers (Obese volunteers supplemented with Ala Before 502.89 µmol/L ± 124.56 *n* = 7 and after 613.87 µmol/L ± 119.43 *n* = 7. Obese volunteers were supplemented with Gln before 415.53 µmol/L ± 19.09 *n* = 13 and after 521.52 µmol/L ± 25.98 *n* = 13 and supplemental data). There was a clear positive correlation between reductions in LPS circulating levels and reductions in waist circumference after glutamine supplementation in overweight and obese subjects (r = 0.629), as shown in Figure 1.

### 3.2. Effects of Glutamine Supplementation on Animal Characteristics

**Figure 2:**
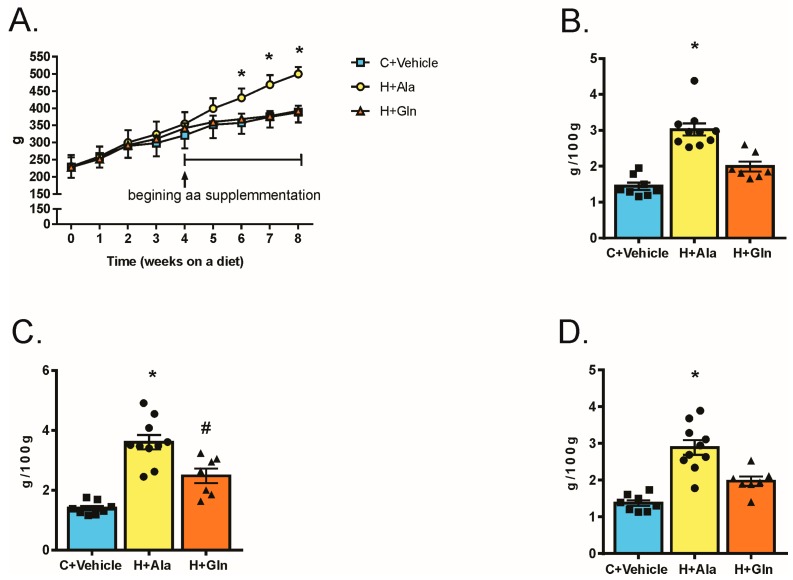
Body Weight (BW) during 8-weeks of amino acids or vehicle (water) supplementation via gavage started at 4-weeks (**A**), epididymal fat mass (**B**), retroperitoneal fat mass (**C**), and mesenteric fat mass (**D**). Rats that received chow diet and vehicle (C+Veh, *n* = 8), rats fed with a high fat diet and supplemented with Alanina (H+Ala, *n* = 10) and rats fed with a high-fat diet and supplemented with glutamine (H+Gln, *n* = 7). Values were displayed as mean ± SEM. Two-Way-ANOVA with a Bonferroni post-test was used in (**A**). One-Way-ANOVA with a Bonferroni post-test that was used in (**B**–**D**). * *p* < 0.05 vs. C+Veh and vs. H+Gln. # *p* < 0.05 vs. C+Veh.

Rats on HFD presented higher body weight than animals that received standard rodent chow (C+vehicle). However, rats on HFD and glutamine supplementation (H+Gln) gain much less weight than rats on HFD treated with alanine (H+Ala) (Figure 2A). As expected, epididymal, retroperitoneal, and mesenteric fat mass was increased in the H+Ala group compared to C+vehicle, but there was a clear decrease in these fat pads in H+Gln groups (Figure 2B–D).

Although rats on HFD had higher (*p* < 0.05) food intake (g) than rats on C+vehicle (24.05 ± 0.79, *n* = 8), there were no differences in food intake between HFD supplemented with glutamine (22.73 ± 0.51, *n* = 7) or alanine (22.65 ± 0.69, *n* = 10).

Related to energy metabolism on HFD rats, glutamine supplementation (32.3 ± 1.7 mL/kg/min, *n* = 4) recovered the reduced O2 consumption (mL/kg/min) observed in the H+Ala group (24.0 ± 1.2, *n* = 5) since the C+ vehicle (35.0 ± 1.0, *n* = 4) displayed similar O2 consumption as the H+Gln group. The RER was reduced (*p* < 0.05) in the H+Ala group (0.72 ± 0.01, *n* = 5) compared to the C+ vehicle group (0.89 ± 0.01, *n* = 4), but the supplementation with glutamine reduced the RER even more significantly, which was significantly lower when compared to the H+Ala group (H+Gln group: 0.79 ± 0.01, *n* = 4 vs. H+Ala group: (0.72 ± 0.01, *n* = 5), which indicates that these animals were oxidizing predominantly fatty acids.

Serum insulin, leptin, TNF-α, LPS and IL-6 levels were higher in the H+Ala group and, after supplementation with glutamine, there was a clear reduction in all these parameters (Table 3). In contrast, adiponectin levels were reduced in the H+Ala group and increased in the H+Gln group (Table 3).

### 3.3. Effect of Glutamine Supplementation on Insulin-Induced Glucose Uptake in Muscle and Adipose Tissue and on the Suppression of Hepatic Glucose Output

**Figure 3:**
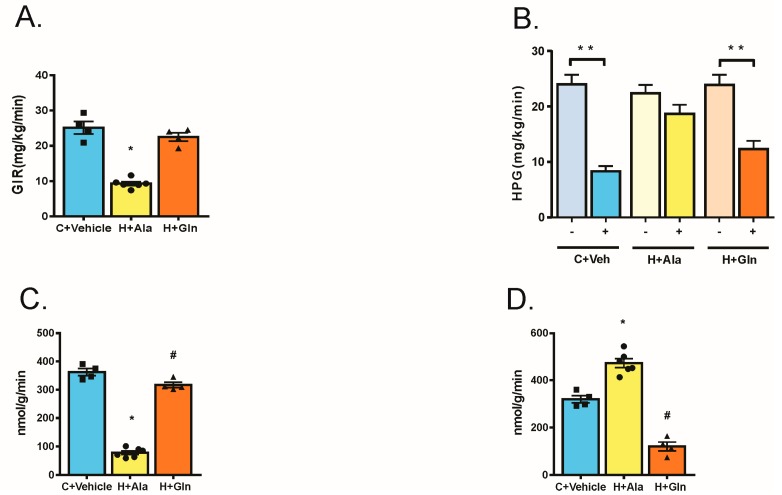
Steady-state glucose infusion rates (GIR) obtained from averaged rates of 90 to 120 min of 10% unlabeled glucose infusion (**A**), insulin-induced suppression of hepatic glucose production (HGP) (**B**), insulin-stimulated muscle glucose uptake (**C**), and insulin-stimulated adipose glucose uptake (**D**). Rats that received the chow diet and vehicle (C+Veh, *n* = 4), rats fed with a high-fat diet and supplemented with Alanina (H+Ala, *n* = 6) and rats fed with a high-fat diet supplemented with glutamine (H+Gln, *n* = 4). Values were displayed as mean ± SEM. One-Way-ANOVA with the Bonferroni post-test was used in A to D. * *p* < 0.05 vs. C+Veh and H+Ala; ** *p* < 0.05 vs. same group without insulin stimulation; # *p* < 0.05 vs. C+Veh.

By using the glucose clamp with tracer infusion, we investigated whole body insulin sensitivity associated with the effect of insulin on hepatic glucose production and glucose uptake in muscle and adipose tissue. The insulin sensitivity, which was determined by the glucose infusion rate (GIR) during the clamp, was diminished in the H+Ala group compared to the other groups (Figure 3). The hepatic glucose production after insulin infusion was less suppressed in the H+Ala group and supplementation with glutamine recovery including the inhibitory effect of insulin on this parameter (Figure 3). Insulin-induced glucose uptake in muscle was reduced in the H+Ala group and normalized in the H+Gln group (Figure 3). In adipose tissue, HFD (H+Ala group) induced an increase in glucose uptake, which confirms previous data that HFD induced insulin resistance in muscle and liver but not in adipose tissue [25,26]. However, the supplementation with glutamine (H+Gln group) dramatically reduced glucose incorporation in adipose tissue to levels even lower than the controls, which indicates clear insulin resistance in this tissue (Figure 3).

### 3.4. Insulin-Induced Insulin Signaling in the Tissues of Rats on HFD Supplemented with Glutamine

**Figure 4:**
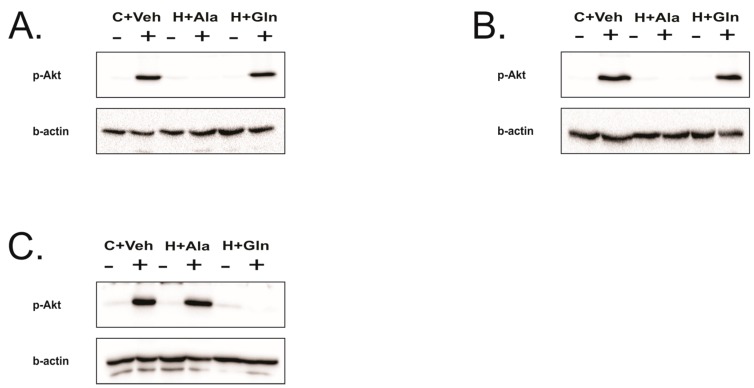
Insulin signaling in the liver, muscle, and adipose tissue. Akt phosphorylation in response to insulin or saline (control) in the liver (**A**) or muscle (**B**) or adipose tissue (**C**) of rats that received the chow diet and vehicle (C+Veh), rats fed with a high-fat diet and supplemented with Alanina (H+Ala), and rats fed with a high-fat diet and supplemented with glutamine (H+Gln). Beta actin was used as the loading control in all tissues. Beta actin was used as loading control in all tissues.

The insulin-induced Akt phosphorylation was reduced in the liver and muscle of rats in the H+Ala group, and glutamine supplementation recovery Akt phosphorylation in these tissues. However, in adipose tissue, insulin-induced Akt phosphorylation, which was increased in the H+Ala group, did not show an increase in the H+Gln group, which indicates that glutamine supplementation prevented increased insulin signaling (Figure 4).

**Figure 5:**
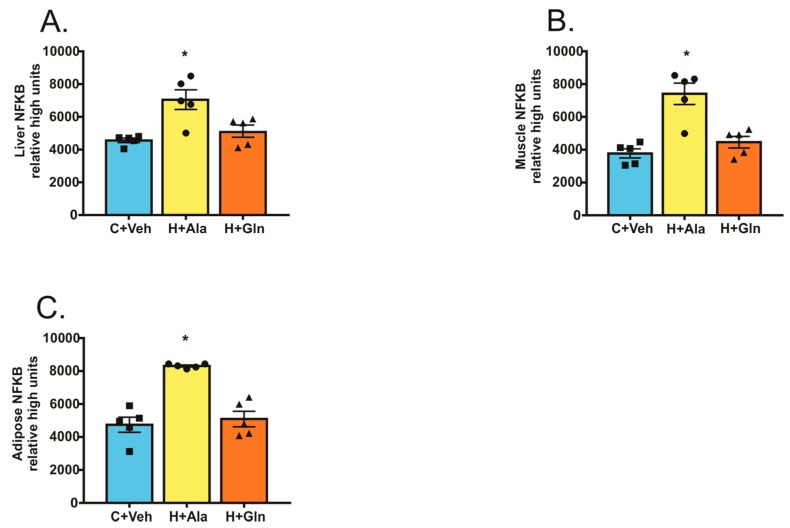
Nuclear factor kappa B (NF-κB) in the liver (**A**), muscle (**B**), and adipose tissue (**C**). Rats that received the chow diet and vehicle (C+Veh, *n* = 5), rats fed with a high-fat diet and supplemented with Alanina (H+Ala; *n* = 5), and rats fed with a high-fat diet and supplemented with glutamine (H+Gln, *n* = 5). Values were displayed as mean ± SEM. One-Way-ANOVA with the Bonferroni post-test was used. * *p* < 0.05 vs. C+Veh and H+Gln.

To investigate whether the reduced LPS circulating levels might contribute to improvements in insulin signaling in the liver and muscle of the H+Gln group, we observed nuclear NF-κB activity in tissues of animals supplemented with glutamine. It is well known that LPS acts by activating Toll-like receptor 4 (TLR4), which induces NF-κB activation. In this regard, the measurement of nuclear NF-κB activity in tissues is an indirect evaluation of TLR4 activation by LPS. The results showed that, in the H+Ala group, NF-κB activity was increased, and the supplementation with glutamine reduced the activity of this nuclear factor in all tissues studied (Figure 5).

**Figure 6:**
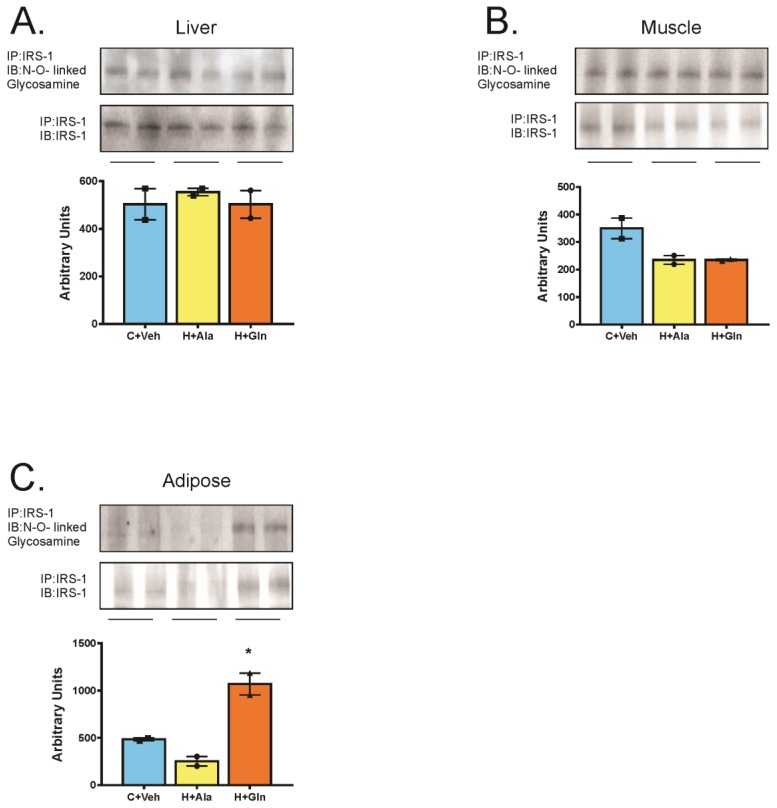
Insulin receptor substrate 1 (IRS1) association with N-*O*-linked glycosamine in the liver (**A**), muscle (**B**), and adipose tissue (**C**). Rats that received a chow diet and vehicle (C+Veh; *n* = 2), rats fed with high-fat diet and supplemented with Alanina (H+Ala; *n* = 2), and rats fed with a high-fat diet and supplemented with glutamine (H+Gln; *n* = 2). Values were displayed as mean ± SEM. One-Way-ANOVA with the Bonferroni post-test was used. * *p* < 0.05 vs. C+Veh and H+Ala.

Multiple mechanisms may account for insulin resistance in obesity, and most of them are linked to inflammatory signaling. Since glutamine reduced the NF-κB activity in adipose tissue, we believe that glutamine-induced insulin resistance in adipose tissue is not related to increased inflammation. In this regard, we next investigated another mechanism of insulin resistance, which is a posttranslational modification of insulin receptor substrates by *O*-linked N-acetylglucosamine (*O*-GlcNAc). We then performed immunoprecipitation of tissue extracts with antibodies anti-IRS-1 followed by blots with antibodies anti *O*-GlcNAc. The results showed that IRS-1 was greater associated with O-GlcNAc only in the adipose tissue of H + Gln group, but not in the liver or muscle. (Figure 6). In parallel, there was a clear reduction in IRS-1 tissue protein levels in adipose tissue of the H+Ala group, which was reversed after glutamine supplementation (Figure 6).

## 4. Discussion

Here, we showed that glutamine supplementation to rats treated with HFD reduces weight gain and improves insulin action and signaling in the liver and muscle. These beneficial effects seem to be associated with tissue-specific insulin resistance in adipose tissue, which prevents the increase in adipose mass in these mice. In a proof of concept study, we also showed that, in humans who are overweight or obese, oral Gln supplementation reduces waist circumference in parallel with a reduction in circulating LPS, which suggests a modulation of microbiota and/or an intestinal barrier.

It is important to mention that we used alanine as a control to give the same amount of calories to the group, which received glutamine. In addition, alanine was chosen because it is the second most abundant circulating amino acid, is produced in muscle and metabolized in the liver as glutamine, and transports ammonia from the muscle to the liver to produce urea [17]. Furthermore, alanine did not significantly affect muscle metabolism or renal and hepatic function [35].

Although in humans, Gln supplementation reduced waist circumference, it did not change body weight and BMI of overweight and obese subjects. The absence of changes on body weight and BMI in humans may be due to the short duration of supplementation. However, waist circumference measurements are a viable and reliable way to measure abdominal fat mass and represent mostly visceral adipose tissue [36,37]. Visceral adipose tissue loss is associated with improved insulin resistance, which decreases the risk of type 2 diabetes development [37,38,39,40].

Both overweight humans and animals on high fat diet with glutamine supplementation reduced circulating lipopolysaccharide levels. The gastrointestinal tract is the main source of LPS, because of its massive bacterial load compared to other anatomical sites [41] and there is a direct and strong association between plasma LPS levels and the degree of intestinal permeability, in different pathological conditions [42]. This translocation of LPS into the plasma leads to binding of LPS to TLR4, which will induce the activation of the NF-κB pathway in different tissues, which results in systemic inflammation [43,44,45,46] and insulin resistance [42]. In mice, the subcutaneous infusion of LPS induces glucose intolerance, insulin resistance, and a very interesting increase in body weight accompanied by increases in adipose tissue [11,47]. The mechanism by which glutamine reduces circulating LPS levels is not completely understood but might involve modulation of intestinal microbiota and or changes in intestinal permeability. Recently, our group demonstrated that L-glutamine altered gut microbiota by decreasing the Firmicutes/Bacteroidetes ratio [21], which is considered a biomarker for obesity. Since we used the same protocol to supplement L-glutamine in the present study, we can, thus, suggest that glutamine may have modulated the microbiota. Taken together, these data with our results suggests that glutamine supplementation may induce changes in intestinal microbiota and in intestinal permeability, which may account for reduced circulating levels of LPS in humans and the animal model, which contributes to improved insulin action.

Previous data demonstrated that in the first months of HFD administration occurred insulin resistance in the liver and muscle, but not in the adipose tissue of rats and mice [25,26]. This tissue-specific protection from insulin resistance in adipose tissue may have an important role in the development of obesity because it allows insulin to increase glucose uptake and synthesize triglycerides in this tissue [7]. However, the inflammatory process that develops in adipose tissue with infiltration of macrophages and the consequent increase in circulating cytokines certainly contribute to worsening the systemic insulin resistance [48,49,50]. In this regard, previous data showed that tissue-specific knockout of the insulin receptor in adipose tissue (FIRKO mice) protects the animal from diet-induced insulin resistance [24]. Based on previous data that glutamine supplementation may induce insulin resistance in adipose cell lines but not in muscle cell lines [27,28,29], we used glutamine supplementation to induce insulin resistance in the adipose tissue of an HFD animal. Our data showed that we could develop a model of adipose tissue-specific insulin resistance in rats by protecting these animals against diet-induced systemic insulin resistance, which is similar to what was described for the FIRKO mice.

We also investigated the molecular mechanism by which glutamine induces insulin resistance only in adipose tissue and reduces glucose uptake and triglycerides synthesis in this tissue. Several mechanisms have been described to explain insulin resistance, and low-grade inflammation represents one of the most important of these mechanisms in obesity [48,49]. However, the adipose insulin resistance induced by glutamine was not related to inflammation, because we found a reduced circulating LPS in humans and the animal model. Furthermore, in adipose tissue of rats, there was a decrease in NF-κB activity, which indicates a clear reduction in an inflammatory milieu. The insulin resistance in adipose tissue of animals on a high fat diet supplemented with glutamine was associated with a reduction of ~50% of fat mass, which suggests that these mechanisms may be linked. One possibility is that GLN increases the activity of the hexosamine pathway (HBP) [51]. GLN is an intermediary substrate to generate glucosamine-6-phosphate via HBP, which is further metabolized to UDP-GlcNAc (UDP-N-acetylglucosamine). UDP-GlcNAc causes posttranslational modification of proteins, which leads to lower lipogenesis and reduced adipose mass [51]. It is well known that the hexosamine pathway can induce insulin resistance through direct post-translational modification of key insulin signaling proteins, via O-linked glycosylation on serine and threonine residues with the GlcNAc moiety [51]. Our results demonstrating increased IRS1/*O*-GlcNAc association in adipose tissue suggest that O-linked GlcNAc post-translational modification may induce insulin resistance in this tissue.

Another important point that should be considered in animals supplemented with glutamine is the increase in adiponectin levels associated with an increase in energy expenditure and a decrease in RER, which indicates that these animals were largely using fatty acids as an energy source. In addition, to act as a glucose-lowering adipokine [52,53,54,55,56,57], adiponectin also increases fatty acids oxidation [58]. Our data showing an increase in circulating adiponectin levels after glutamine supplementation may explain the increased energy expenditure and fat oxidation, which reinforces the protection from diet-induced obesity in these animals.

Our study has some limitations such as the short time of glutamine supplementation in humans and the absence of a gold standard method to measure insulin sensitivity, and of an oral glucose tolerance test in humans. In addition, 24-hour dietary recall and our assessment of the physical activity level have limitations.

Our data show that glutamine supplementation, in animals and humans, was accompanied by an increase in plasma glutamine levels and in rat tissue by an increase in IRS1/*O*-GlcNAc in adipose tissue but not in the liver and the muscle tissue. This action of glutamine mainly in adipose tissue may be a consequence of differential mechanisms involved in the glutamine transport among tissues [59], but, certainly, this point deserves a deep investigation.

## 5. Conclusions

In summary, our data showed that glutamine supplementation could reduce the insulin action and glucose uptake in fat and adipose mass, which improves the insulin action and signaling in the liver and muscle of rats. In addition, a proof of concept study showed that, in humans, glutamine supplementation for a short time is accompanied by a reduction in waist circumference, in circulating LPS. Our preliminary data suggest that further investigations with glutamine supplementation should be performed for longer periods. In addition, it would be interesting to investigate whether glutamine is also able to induce insulin resistance in the adipose tissue of humans, which may have some long-term detrimental consequences.

## Figures and Tables

**Table 1:** Differences in metabolic characteristics of overweight volunteers before and after supplementation with glutamine or alanine.

	Overweight Volunteers			
	Ala Suppl Difference between After and Before (*n* = 8)	*p*-Value	Gln Supplemental Difference between After and Before (*n* = 11)	*p*-Value
Height (m)				
Weight (kg)	0.4 ± 0.74	0.17	−0.44 ± 0.89	0.13
BMI (kg/m^2^)	0.16 ± 0.29	0.16	−0.17 ± 0.32	0.10
Serum amino acid levels (µmol/L)	106.36 ± 12.55	0.0001	111.20 ± 13.83	0.0001
WC (cm)	−0.19 ± 0.8	0.52	−1.82 ± 1.4	0.001 *
Glucose (mmol/L)	−0.08 ± 0.21	0.34	0.06 ±0.6	0.83
Insulin (μU/mL)	0.16 ± 0.74	0.33	−1.00 ± 2.89	0.41
TNF-α (pg/mL)	0.04 ± 0.96	0.91	−2.88 ± 3.94	0.28
IL-1β (pg/mL)	0.01 ± 0.17	0.84	−0.14 ± 0.33	0.31
IL-6 (pg/mL)	−0.006 ± 0.38	0.96	0.271 ± 1.413	0.53
LPS (EU/mL)	−0.001 ± 0.006	>0.99	−0.041 ± 0.059	0.04 *

Gln suppl, L-glutamine supplementation. Ala suppl, alanine supplementation. BMI, body mass index. WC, waist circumference. TNF-α, tumor necrosis alpha. IL-1β, interleukin-1 beta. IL6, interleukin 6. LPS, lipopolysaccharide. Data were expressed as the difference between before and after 14 days of supplementation with either Ala or Gln. Serum parameters were obtained from overnight fasted volunteers. Data were expressed as mean ± SD. *p*-value indicates the difference between before and after glutamine or alanine supplementation obtained by paired Student’s *t* test under normality and the Wilcoxon Mann–Whitney test otherwise. * *p* indicates a significant difference before the same amino acid supplementation.

**Table 2:** Differences in metabolic characteristics of obese volunteers before and after supplementation with glutamine or alanine.

	Obese Volunteers			
	Ala Suppl Difference between After and Before (*n* = 7)	*p*-Value	Gln Supplemented Difference between After and Before (*n* = 13)	*p*-Value
Height (m)				
Weight (kg)	0.3 ± 1.02	0.46	−0.21 ± 0.76	0.34
BMI (kg/m^2^)	0.12 ± 0.39	0.44	−0.08 ± 0.30	0.37
Serum amino acid levels (µmol/L)	110.97 ± 15.53	0.0001	105.99 ± 14.99	0.0001
WC (cm)	0.0 ± 1.19	>0.99	−2.61 ± 2.21	0.002 *
Glucose (mmol/L)	0.25 ± 0.28	0.06	0.03 ± 0.41	0.84
Insulin (μU/mL)	0.34 ± 0.94	0.27	−1.15 ± 1.7	0.037 *
TNF-α (pg/mL)	0.11 ± 0.63	0.67	−2.65 ± 4.18	0.09
IL-1β (pg/mL)	0.06 ± 0.45	0.74	−0.24 ± 0.74	0.41
IL-6 (pg/mL)	0.07 ± 0.21	0.37	−0.27 ± 0.74	0.22
LPS (EU/mL)	−0.001 ± 0.01	0.78	−0.08 ± 0.12	0.08

Gln suppl, L-glutamine supplementation. Ala suppl, alanine supplementation. BMI, body mass index. WC, waist circumference. TNF-α, tumor necrosis alpha. IL-1β, interleukin-1 beta. IL6, interleukin 6. LPS, lipopolysaccharide. Data were expressed as the difference between before and after 14 days of supplementation with either Ala or Gln. Serum parameters were obtained from volunteers who fasted overnight. Data were expressed as mean ± SD. The *p*-value indicates the difference between before and after glutamine or alanine supplementation obtained by paired Student’s *t* test under normality and Wilcoxon Mann–Whitney test otherwise. * *p* indicates a significant difference before the same amino acid supplementation.

**Table 3:** Animal’s metabolic characteristics.

	C+vehicle	H+Ala	H+Gln
Insulin (ng/mL)	0.24 ± 0.01 (*n* = 5)	1.14 ±0.21 * (*n* = 8)	0.49 ±0.08 # (*n* = 6)
Adiponectin (μg/mL)	8.16 ± 0.29 (*n* = 5)	4.86 ± 0.22 * (*n* = 6)	6.78 ± 0.4 # (*n* = 6)
TNF alpha (pg/mL)	95.94 ± 7.06 (*n* = 4)	499.60 ± 63.23 * (*n* = 5)	250.00 ± 34.86 (*n* = 5)
IL6 (pg/mL)	349.50 ± 5.80 (*n* = 4)	609.50 ± 46.10 * (*n* = 5)	347.50 ± 12.90 (*n* = 4)
LPS (EU/mL)	0.464 ± 0.05 (*n* = 5)	1.15 ± 0.16 * (*n* = 6)	0.74 ± 0.06 (*n* = 6)

Data were expressed means ± SEM. * *p* < 0.05 vs. C+vehicle group and H+Gln; # *p* < 0.05 vs. C+vehicle group. One-Way-ANOVA with the Bonferroni post-test was used for statistical analysis. GIR, steady-state glucose infusion rates were obtained from averaged rates of 90–120 min of 10% unlabeled glucose infusion. HGP, hepatic glucose production. TNF alpha, tumor necrosis alpha. IL-1 beta, interleukin-1 beta. IL-6, interleukin 6; LPS, lipopolysaccharide.

## Supplementary Materials

The following are available online at https://www.mdpi.com/2072-6643/11/3/536/s1, Table S1: Metabolic characteristics of overweight volunteers before and after supplementation with glutamine or alanine. Table S2: Metabolic characteristics of obese volunteers before and after supplementation with glutamine or alanine.
