# Supplementary material for: Oral Glutamine Supplementation Reduces Obesity, Pro-Inflammatory Markers, and Improves Insulin Sensitivity in DIO Wistar Rats and Reduces Waist Circumference in Overweight and Obese Humans
Source: Nutrients. 2019 Mar 1;11(3):536. doi: 10.3390/nu11030536 (PMC6471297; doi:10.3390/nu11030536)
Supplement: Supplementary file 1 [file nutrients-11-00536-s001.pdf]

Supplemental Data

Table S1. Metabolic characteristics of overweight volunteers before and after supplementation with glutamine or alanine.

	Overweight Volunteers					
	**Ala Suppl (*n* = 8)**	**Ala Suppl (*n* = 8)**		**Gln Suppl (*n* = 11)**	**Gln Suppl (*n* = 11)**	
	Before	After	*p*-Value	Before	After	*p*-Value
Height (m)	1.63 ± 0.05	1.63 ± 0.05		1.62 ± 0.08	1.62 ± 0.08	
Weight (kg)	73.15 ± 6.51	73.55 ± 6.30	0.17	73.8 ± 8.65	73.34 ± 8.89	0.13
BMI (kg/m^2)	27.6 ± 1.80	27.75 ± 1.76	0.16	28.2 ± 1.09	28.00 ± 1.15	0.10
Serum amino acid levels (μmol/L)	448.09 ± 52.30	554.46 ± 57.64	0.0001	419.88 ± 35.11	531.09 ± 36.52	0.0001
WC (cm)	85.5 ± 4.84	85.3 ± 4.51	0.52	87 ± 5.71	85.18 ± 5.66	0.001 *
Glucose (mmol/L)	4.73 ± 0.37	4.65 ± 0.40	0.34	4.86 ± 0.58	4.92 ± 0.42	083
Insulin (μU/mL)	2.78 ± 1.74	3.35 ± 1.35	0.33	5.53 ± 0.98	4.97 ± 1.96	0.41
TNF-α (pg/mL)	3.46 ± 1.92	3.50 ± 1.75	0.91	6.37 ± 4.22	3.56 ± 1.25	0.28
IL-1β (pg/mL)	2.80 ± 0.80	2.81 ± 0.70	0.84	3.85 ± 0.33	3.71 ± 0.20	0.31
IL-6 (pg/mL)	1.80 ± 0.67	1.79 ± 0.62	0.96	1.33 ± 0.47	1.60 ± 1.15	0.53
LPS (EU/mL)	0.59 ± 0.07	0.59 ± 0.07	>0,99	0.51 ± 0.08	0.47 ± 0.05	0.04 *

Gln suppl, L-glutamine supplementation; Ala suppl, alanine supplementation; BMI, body mass index; WC, waist circumference; TNF-α, tumor necrosis alpha; IL-1β, interleukin-1 beta; IL6, interleukin 6; LPS, lipopolysaccharide. Data were collected before and after 14 days of supplementation with either Ala or Gln. Serum parameters were obtained from overnight fasted volunteers. Data were expressed as mean ± SD. *p*-value indicates the difference between before and after glutamine or alanine supplementation obtained by paired Student's t test under normality and Wilcoxon Mann–Whitney test otherwise. * *p* indicates a significant difference between before the same amino acid supplementation.

Table S2. Metabolic characteristics of obese volunteers before and after supplementation with glutamine or alanine.

	Obese Volunteers					
	**Ala Suppl (*n* = 7)**	**Ala Suppl (*n* = 7)**		**Gln Suppl (*n* = 13)**	**Gln Suppl (*n* = 13)**	
	**Before**	**After**	***p*-value**	**Before**	**After**	***p*-value**
Height (m)	1.61 ± 0.086	1.61 ± 0.086		1.58 ± 0.06	1.58 ± 0.06	
Weight (kg)	86.6 ± 12.69	86.93 ± 12.45	0.46	86.19 ± 13.89	85.98 ± 13.82	0.34
BMI (kg/m^2)	33.07 ± 2.97	33.19 ± 2.91	0.44	34.51 ± 5.10	34.42 ± 5.08	0.37

Serum amino acid levels (μmol/L)	502.89 ± 124.56	613.87 ± 119.43	0.0001	415.53 ± 19.09	521.52 ± 25.98	0.0001
WC (cm)	95.1 ± 5.57	95.14 ± 5.86	>0.99	96.07 ± 9.61	93.46 ± 9.06	0.002 *
Glucose (mmol/L)	4.82 ± 0.47	5.07 ± 0.65	0.06	4.76 ± 0.47	4.78 ± 0.55	0.84
Insulin (μU/mL)	5.01 ± 3.03	5.40 ± 3.26	0.27	5.56 ± 4.27	4.41 ± 3.78	0.037*
TNF-α (pg/mL)	3.08 ± 1.39	3.18 ± 1.16	0.67	5.91 ± 3.54	3.04 ± 1.25	0.09
IL-1β (pg/mL)	3.03 ± 0.77	3.09 ± 0.76	0.74	3.42 ± 0.79	3.18 ± 0.71	0.41
IL-6 (pg/mL)	3.12 ± 3.998	3.20 ± 3.92	0.37	2.30 ± 1.07	2.01 ± 0.80	0.22
LPS (EU/mL)	0.58 ± 0.05	0.58 ± 0.06	0.78	0.56 ± 0.13	0.47 ± 0.06	0.08

Gln suppl, L-glutamine supplementation; Ala suppl, alanine supplementation; BMI, body mass index; WC, waist circumference; TNF-α, tumor necrosis alpha; IL-1β, interleukin-1 beta; IL6, interleukin 6; LPS, lipopolysaccharide. Data were collected before and after 14 days of supplementation with either Ala or Gln. Serum parameters were obtained from overnight fasted volunteers. Data were expressed as mean ± SD. *p*-value indicates the difference between before and after glutamine or alanine supplementation obtained by paired Student's t test under normality and Wilcoxon Mann–Whitney test otherwise. * *p* indicates a significant difference between before the same amino acid supplementation.
